# Involvement of the Clock Gene *Rev-erb alpha* in the Regulation of Glucagon Secretion in Pancreatic Alpha-Cells

**DOI:** 10.1371/journal.pone.0069939

**Published:** 2013-07-25

**Authors:** Elaine Vieira, Laura Marroquí, Ana Lucia C. Figueroa, Beatriz Merino, Rebeca Fernandez-Ruiz, Angel Nadal, Thomas P. Burris, Ramon Gomis, Ivan Quesada

**Affiliations:** 1 Instituto de Bioingeniería, Universidad Miguel Hernandez de Elche, Elche, Spain; 2 CIBER de Diabetes y Enfermedades Metabólicas Asociadas, Barcelona, Spain; 3 Diabetes and Obesity Laboratory, Institut d'investigacions Biomèdiques August Pi i Sunyer, Barcelona, Spain; 4 Endocrinology and Diabetes Unit, Hospital Clinic, Universitat de Barcelona, Barcelona, Spain; 5 Department of Molecular Therapeutics, The Scripps Research Institute, Jupiter, Florida, United States of America; University of Santiago de Compostela School of Medicine - CIMUS, Spain

## Abstract

Disruption of pancreatic clock genes impairs pancreatic beta-cell function, leading to the onset of diabetes. Despite the importance of pancreatic alpha-cells in the regulation of glucose homeostasis and in diabetes pathophysiology, nothing is known about the role of clock genes in these cells. Here, we identify the clock gene *Rev-erb alpha* as a new intracellular regulator of glucagon secretion. *Rev-erb alpha* down-regulation by siRNA (60–70% inhibition) in alphaTC1-9 cells inhibited low-glucose induced glucagon secretion (p<0.05) and led to a decrease in key genes of the exocytotic machinery. The *Rev-erb alpha* agonist GSK4112 increased glucagon secretion (1.6 fold) and intracellular calcium signals in alphaTC1-9 cells and mouse primary alpha-cells, whereas the *Rev-erb alpha* antagonist SR8278 produced the opposite effect. At 0.5 mM glucose, alphaTC1-9 cells exhibited intrinsic circadian *Rev-erb alpha* expression oscillations that were inhibited by 11 mM glucose. In mouse primary alpha-cells, glucose induced similar effects (p<0.001). High glucose inhibited key genes controlled by AMPK such as Nampt, Sirt1 and PGC-1 alpha in alphaTC1-9 cells (p<0.05). AMPK activation by metformin completely reversed the inhibitory effect of glucose on Nampt-Sirt1-PGC-1 alpha and *Rev-erb alpha*. Nampt inhibition decreased Sirt1, PGC-1 alpha and *Rev-erb alpha* mRNA expression (p<0.01) and glucagon release (p<0.05). These findings identify *Rev-erb alpha* as a new intracellular regulator of glucagon secretion via AMPK/Nampt/Sirt1 pathway.

## Introduction

Numerous biological processes such as body temperature, sleep/wake cycle, feeding, metabolism and hormone release display 24 hours rhythms that are driven by cell circadian clocks [1], [2]. In mammals, the central pacemaker of the clock machinery is located in the hypothalamus, more precisely in neurons of the suprachiasmatic nuclei. Besides the central location in the brain, peripheral molecular clocks exist in several organs, including liver, kidneys, muscle, adipose tissue and pancreas [3], [4], [5], [6]. The central and peripheral oscillators share a common molecular circuitry, with a battery of transcriptional activators and repressors forming a self-sustained transcriptional feedback loop. The primary loop is composed by the transcription factors CLOCK (circadian locomotor output cycles kaput) and BMAL1 (brain and Muscle arnt-like 1) which drive the transcription of the Per1 (period homolog drosophila 1) and Per2 (period homolog drosophila 2) and Cry1 (cryptochrome 1) Cry2 (cryptochrome 2) genes [7]. PER and CRY inhibit their own CLOCK: BMAL1-induced transcription, and turnover of PER and CRY allows this cycle to continue. Important nuclear receptors such as *Rev-erb alpha* (reverse-eritroblastosis virus alpha, nuclear receptor encoded by NR1D1) can also regulate CLOCK and BMAL1 expression. Besides its role in the control of the molecular clock, *Rev-erb alpha* has also been shown to regulate lipid metabolism and bile acid homeostasis in the liver [8], [9], adipogenesis [10] gluconeogenic genes [11], [12], as well as insulin secretion [13]. Thus, *Rev-erb alpha* is considered a good candidate to link circadian rhythms and metabolism.

Disturbances in the regulation of circadian rhythms have been implicated in the development of metabolic disorders such as obesity and type 2 diabetes. For instance, CLOCK and BMAL1 disruption leads to alterations in the expression of beta-cell genes involved in growth, survival and synaptic vesicle assembly, which can trigger the onset of diabetes [14]. The regulation of glucagon secretion in response to glucose plays an essential role in the control of glycaemic levels. Alteration of the alpha-cell normal function is part of the events that are present in the pathophysiology of diabetes mellitus [15]. Actually, hyperglucagonemia is typically found in diabetic patients, favoring hepatic gluconeogenesis and hyperglycemia. Despite its importance, little is known about the mechanisms that control glucose-dependent alpha-cell glucagon release, particularly those that are involved in the coupling of plasma glucose levels with alpha-cell metabolism and exocytosis.

One of the molecular pathways by which glucose regulates glucagon secretion is through the AMP-activated protein kinase (AMPK) [16]. Interestingly, AMPK has been shown to link metabolism and the Clock machinery. For instance, the AMPK-Nampt (nicotinamide phosphoribosyltransferase)-Sirt1 (silent mating type information regulation 1 homolog) pathway has been shown to change the core clock proteins in white adipose tissue [17]. In skeletal muscle, AMPK activation changes the expression pattern of clock genes and metabolism via AMPKγ3 [5]. Since AMPK is an important mediator of glucagon secretion and can also modulate several clock components, we decided to study the role of *Rev-erb alpha* in pancreatic alpha-cell glucagon secretion and the potential involvement of AMPK in this process. Here, we showed that the clock gene *Rev-erb alpha* is present in the pancreatic alpha-cell, is glucose-modulated and participates in the regulation of glucagon release in response to extracellular glucose changes through the AMPK-Nampt-Sirt1 pathway. Thus, the present work identifies the clock gene *Rev-erb alpha* as an important intracellular player in the control of pancreatic alpha-cell function.

## Materials and Methods

### Ethics Statement

All animal work has been conducted according to national and international guidelines. All protocols were approved by the ethical committee of Miguel Hernandez University “Comisión de Ética en la Investigación Experimental” and specifically reviewed and approved this study (approval ID: IB-IQM-001-10).

### Cell Culture

The glucagon-releasing alphaTC1-9 cells were purchased from American Type Cultures Collection (ATCC CRL-2350; Barcelona, Spain). They were grown in DMEM (Invitrogen, Barcelona, Spain) supplemented with 4 mM L-glutamine, 16 mM glucose, 19 mM NaHCO3, 10% FBS, 15 mM HEPES and 0.1 mM non-essential amino acids. To measure circadian *Rev-erb alpha* gene expression in vitro, we performed a short treatment with 50% serum (serum shock) to the confluent, serum-starved alphaTC1-9 cells, according to previous studies [18], [19]. After 2 hours of serum shock, the medium was changed to DMEM serum-free medium and RNA was extracted every 6 hours during 48 hours for gene expression measurements. The experiments were done in serum-free medium to discriminate between oscillations in cell cycle and intrinsic circadian oscillations [18], [19]. Cell viability was performed in alphaTC1-9 cells in presence of 0.5 mM glucose and 11 mM glucose for 24 and 48 h using Trypan Blue stain 0.4% (Life technologies Ltd, Paisley, UK) and measured with Countless® automated cell counter (Invitrogen, Life technologies Ltd, Paisley, UK).

### Islet Isolation and Ca^2+^ Measurements

All protocols were approved by our animal Care Committee according to national regulations (ethical number: IB-IQM-001-10). Adult C57BL/6J mice were sacrificed at 8 weeks old by cervical dislocation and islets were then isolated by collagenase digestion [20] and hypothalamus and liver were collected for gene expression measurements. Mice were kept under 12 h:12 h light dark cycle (lights on at 07∶00 and lights off at 19∶00) with food *ad libitum*. For Ca^2+^ experiments, islets were dispersed into single cells in a Ca^2+^-deficient medium containing 0.5 mM EDTA and 0.05% trypsin followed by brief shaking. Then, cells were cultured overnight at 37°C in RPMI 1640 (Sigma, Madrid, Spain) supplemented with 10% fetal calf serum, 100 IU/ml penicillin, 0.1 mg/ml streptomycin and 11 mM D-glucose [21]. Either alphaTC1-9 cells or isolated mouse islet cells were loaded with Fura-2 (5 µM) for 1 hour at room temperature. Cells were placed on a perfusion chamber mounted on the microscope stage and perfused at a rate of 1.5 ml/min with a modified Ringer solution containing (in mM): 120 NaCl, 5 KCl, 25 NaHCO3, 1.1 MgCl2 and 2.5 CaCl2; pH = 7.4, gassed with 95% O2 and 5% CO2. Calcium signals were recorded using an inverted epifluorescence microscope (Zeiss, Axiovert 200) and a Hamamatsu Digital Camera C4742-95 (Hamamatsu Photonics, Barcelona, Spain). Fluorescence records were expressed as the ratio of fluorescence (R) at 340 nm and 380 nm (F340/F380). We analyzed the area-under-the-curve (AUC) of the whole period of stimulation using Origin software (Origin, Origin Lab Corporation, MA USA). This parameter is an indicator of the global calcium increase in the cells [22]. As previously reported, alpha-cells were identified by their spontaneous calcium signalling activity at low glucose concentrations and their characteristic response to epinephrine [13], [23], [24].

### Glucagon Secretion and Content

Cells were cultured for 2 days in DMEM prior to the measurements of glucagon secretion. Then, cells were incubated at 37°C in 0.5 ml Krebs–Ringer bicarbonate buffer supplemented with 15 mM HEPES, 0.5% BSA and 5.6 mM glucose, pH 7.4 for 30 min. Afterward, batches of 1×10^6^ alphaTC1-9 cells were incubated for 1 h at 37°C in 0.5 ml of Krebs–Ringer bicarbonate buffer supplemented with 0.5 mM, 11.2 mM glucose, the *Rev-erb alpha* agonist GSK4112 (Sigma St Louis, USA), Hemin (Sigma St Louis, USA), the *Rev-erb alpha* antagonist SR8278 (Sigma St Louis, USA) or the Nampt inhibitor FK866 (Cayman Chemical, Ann Arbor, USA). The supernatant was collected and glucagon secretion was measured. Cells were then lysed with 50 µl of lysis buffer (70% ethanol, 0.4% HCl at 30%, 24.6% distilled water) and incubated overnight at 4°C. Samples were centrifuged at 2500 rpm for 5 min and the supernatant was collected for glucagon content analysis. Glucagon was detected by ELISA using a commercial kit (YK090; Gentaur, Brussels, Belgium). Total protein was determined by the Bradford method.

### Quantitative Real-time PCR

Quantitative PCR assays were performed using CFX96 Real Time System (Biorad, Hercules, USA). Reactions were carried out in a final volume of 10 µl, containing 200 nM of each primer, 100 nM of endogenous control primer, 1 µl of cDNA and IQ™ Sybr® Green Supermix (Biorad, Hercules, USA). Samples were subjected to the following conditions: 10 min at 95°C, 40 cycles (10 s 95°C/7 s 60°C/12 s 72°C), and a melting curve of 63 to 95°C with a slope of 0.1°C/s. The housekeeping gene *rplp0* (ribosomal protein large P0, *alias 36B4*) was used as the endogenous control for quantification [5]. The results were analyzed with CFX Manager Version 1.6 (Biorad, Hercules, USA) and values were expressed as the relative expression respect to control levels (2^−ΔΔct^). Primers sequences are described in Table 1.

**Table 1:** Quantitative Real-Time PCR primers.

NAME	Accession No.	Sense Primer (5′-3′)	Antisense primer (5′-3′)
Rplp0	NM_007475	GAGGAATCAGATGAGGATATGGGA	AAGCAGGCTGACTTGGTTGC
Vamp3	NM_009498	CTCACCAAGGCATCAGTCTG	ATTCTAAGAGCACCAGGCATC
Munc18	NM_001113569	GGACTG AAG AGC GTC GTG TG	TTG GTG GTA AAC TCA TCT AAC AGC
SNAP25	NM_011428	GTC TTT CCT TCC CTC CCT ACC	AGT CAG TGG TGC TTC GTT AAA
Syntaxin 1a	NM_016801	GATGAG AAGACAAAG GAGGAACTG	ATG AGC GGT TCA GAC CTT CC
Nampt	NM_021524.2	GAGACTGCCGGCATAGGGGC	GGTACTGTGCTCTGCCGCTGG
Sirt1	NM_001159589.1	CCTGACTTCAGATCAAGAGA	TGTCTCCACGAACAGCTTCA
PGC1alpha	NM_0089042	AGCCGTGACCACTGACAACGAG	GCTGCATGGTTCTGAGTGCTAAG
Rev-erb alpha	NM_145434	GGTGCGCTTTGCATCGTT	GGTTGTGCGGCTCAGGAA
Clock	NM_007715	TTGCTCCACGGGAATCCTT	GGAGGGAAAGTGCTCTGTTGTAG
Per2	NM_011066	ATGCTCGCCATCCACAAGA	GCGGAATCGAATGGGAGAAT
Cry1	NM_007771	CTGGCGTGGAAGTCATCGT	CTGTCCGCCATTGAGTTCTATG
Cry2	NM_009963	AGCCCAGGCCAAGAGGAA	GTTTTTCAGGCCCACTCTACCTT
Bmal1	NM_007489	GGACTTCGCCTCTACCTGTTCA	AACCATGTGCGAGTGCAGGCGC
Glucagon	NM_008100	GGCTCCTTCTCTGACGAGATGAGCAC	CTGGCACGAGATGTTGTGAAGATGG
Alas-1	NM_020559	ATCATCTCTGGGACGCTTGGTA	GGACGGTGTCGATCAGCAA

### Interference RNA

SiRNA treatment was performed in alphaTC1-9 cells as previously described [25], [26]. Cells were transfected overnight with 50 nM of siRNAs Silencer® Pre-designed *Rev-erb alpha* (Ambion, TX,USA) or 50 nM of Silencer® labelled negative control #2 siRNA (Ambion, TX, USA) in optiMEM® I (Invitrogen, Carlsbad, USA) culture medium without antibiotics and 1% of Lipofectamine 2000 (Invitrogen, Carlsbad, USA). The following *Rev-erb alpha* siRNA sequences were used (5′-3′): GCAUCGUUGUUCAACGUGAtt, sense; UCACGUUGAACAACGAUGCaa, antisense. After overnight incubation, the transfection medium was replaced by DMEM culture medium for 24 h before the start of the experiments.

### Western-blot Analysis

Cell pellets were obtained by centrifuging at 1000×*g* for 10 min and resuspended in 50 µl of lysis buffer (Cell Signaling Technology, Danvers, MA). Cell extracts were subjected to sodium dodecyl sulfate (SDS) polyacrylamide gel electrophoresis (Mini-Protean®TGX™ Precast Gel, 4–20% gels, Biorad). Pre-stained SDS-PAGE standards were included for molecular mass estimation. The transfer to PVDF membranes was performed at 125 mA for 90 min in a buffer with 2.5 mM Tris base, 9 mM glycine, 20% methanol. After membranes were blocked with 2% non-fat dry milk, they were incubated with the following antibodies: rabbit polyclonal anti-actin (1∶1000; Sigma, Saint Louis, USA), anti-Rev-erb alpha (1∶500; Abcam, Cambridge, UK), pAMPK (Thr^172^) (1∶1000; Abcam, Cambridge, UK) and Nampt antibody (1∶1000; Abcam, Cambridge, UK). Membranes were incubated with appropriate HRP-conjugated antibodies (Biorad, Hercules, USA). Protein bands were revealed by using the ECL western blot substrate (Thermo Fisher Scientific, Madrid, Spain). Intensity of the bands was quantified using Scion image software (Frederick, MD USA).

### Cell Sorting

Isolated cells were placed in sorting buffer containing 1% BSA, 2.5 mM glucose, Ca^2+^, Mg^2+^ free PBS, 1 mM EDTA, 25 mM Hepes and transferred to a fluorescence activated cell sorter (FACS; BD FACSARIA SORP) equipped with 488 nm and 355 nm. The FACS was used to separate pancreatic beta-cells and alpha cells by cellular autofluorescence as recently reported by Kohler [27].

### Immunohistochemistry

After sorting approximately 10.000 cells from each cell fraction were subjected to cytospin in order to perform immunofluorescence analysis. Cells are fixed in slides with Paraformaldehyde 4% and permeabilized with triton 0.2% for 45 minutes. Blocking was then performed with PBS-BSA 0.1% for 30 minutes at room temperature. Cells were incubated overnight at 4°C with insulin and glucagon antibodies (DAKO), and subsequently incubated with secondary antibodies Cy3 antiguineapig for insulin and Cy2 antirabbit for glucagon (Jackson Immunoresearch) and washed with PBS. Analysis performed with Image J program. Alpha-cell enriched fraction was composed of 80% alpha-cells and 4% beta-cells while the beta-cell enriched fraction had 75% beta-cells and no alpha-cells.

### Statistical Analysis

Data is shown as mean ± SEM. Student’s *t* test, one-way ANOVA or two-way ANOVA with Bonferroni correction were performed as appropriate with a level of significance p<0.05.

## Results

### Glucose Regulates Rev-erb Alpha Gene and Protein Expression in alphaTC1-9 Cells and in Mouse Pancreatic Alpha Cells

**Figure 1:**
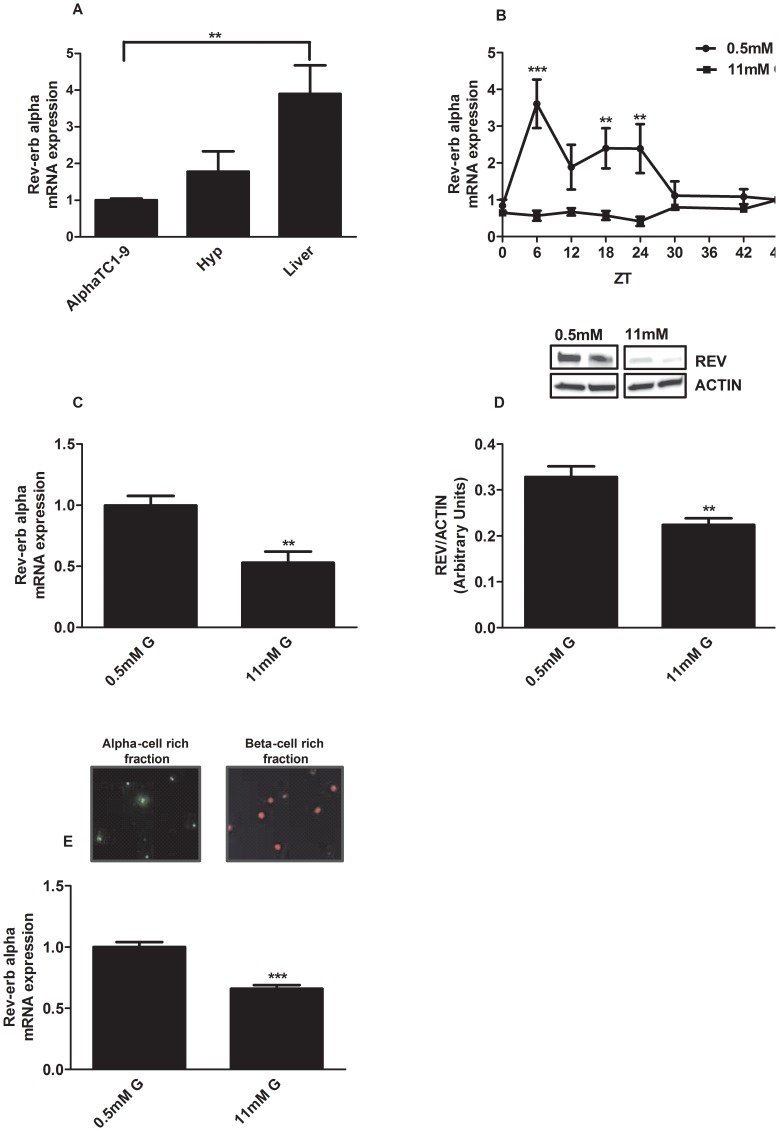
Glucose downregulates Rev-erb alpha gene expression in alphaTC1-9 cells and mouse pancreatic alpha cells. (**A**) Gene expression of *Rev-erb alpha* in glucagon-secreting alphaTC1-9 cells, hypothalamus and liver (n = 5) The statistical significance was performed comparing the expression levels in alphaTC1-9 cells with the other tissues. (**B**) Intrinsic oscillations of *Rev-erb alpha* gene expression during 48 hours. AlphaTC1-9 cells were treated with 0.5 mM glucose (circles) and 11 mM glucose (square) for 48 hours (n = 6). The statistical significance was performed between the ZT (Zeitgeber Time) times comparing 0.5 mM and 11 mM. (**C**) *Rev-erb alpha* gene expression in alphaTC1-9 cells treated with 0.5 mM glucose and 11 mM glucose at ZT6 (n = 4–5). (**D**) Rev-erb alpha protein expression in alphaTC1-9 cells treated with 0.5 mM glucose and 11 mM glucose at ZT6 (n = 4). (**E**) *Rev-erb alpha* gene expression in primary mouse alpha-cells separated by FACS sorting and treated with 0.5 mM glucose and 11 mM glucose at ZT6 (n = 4). Alpha-cell enriched fraction: 80% alpha cells and 4% beta cells. Beta-cells enriched fraction: 75% beta-cells and no alpha-cells *p<0.05, **p<0.01, *** p<0.001. Data are expressed as mean ±S.E.M.

To analyze the mRNA expression of several clock genes, we used the mouse alpha-cell line alphaTC1-9 which has been validated as a good model to study alpha cell function [16], [25], [28]. The mRNA levels in this cell line were compared with those of the hypothalamus and liver. *Rev-erb alpha* expression in alphaTC1-9 cells was comparable with that in the hypothalamus whereas the expression levels in the liver were much higher (Fig. 1A). The expression of Clock, Bmal1 and Cry1 in alphaTC1-9 was similar to that in the hypothalamus and liver (S 1). Per-1 expression was lower in the liver compared with alphaTC1-9 cells whereas Per2 and Cry2 had lower expression levels in the hypothalamus (S 1). To study whether alpha-cells exhibit an oscillatory pattern of *Rev-erb alpha* expression along the day, we next performed mRNA measurements every 6 hours during a 48 h, as previously described [19]. To check that culture conditions were not affecting cell viability, we measured this parameter at 0.5 and 11 mM glucose after 24 and 48 hours of incubation. No differences were found in cell viability between both glucose concentrations in any of the time points measured (data not shown). Figure 1B shows that *Rev-erb alpha* mRNA levels oscillated in vitro at low glucose concentrations (0.5 mM) with an expression peak at ZT6 (Zeitgeber Time). Interestingly, in our experiments high glucose concentrations (11 mM) inhibited *Rev-erb alpha* expression levels. To further confirm the glucose effect on *Rev-erb alpha* at both the mRNA and protein levels, we performed similar experiments at ZT6. As expected, high glucose concentrations produced an inhibitory action on both *Rev-erb alpha* gene (Fig. 1C) and protein expression (Fig. 1D). We next studied the glucose effect at ZT6 in sorted mouse primary alpha-cells. In agreement with the results in alphaTC1-9 cells, high glucose concentrations down-regulated the *Rev-erb alpha* mRNA levels compared to the effect of 0.5 mM glucose in the alpha cell rich fraction of the primary isolated islet cells (Fig. 1E). These findings indicate that both alphaTC1-9 cells and mouse pancreatic alpha-cells express *Rev-erb alpha* and that glucose regulates the expression of this clock gene. Additionally, we show that alphaTC1-9 cells exhibit intrinsic circadian oscillations of *Rev-erb alpha,* indicating that alpha-cells of the endocrine pancreas have their own biological clock.

### Rev-erb Alpha Regulates Glucagon Secretion in alphaTC1-9 Cells

**Figure 2:**
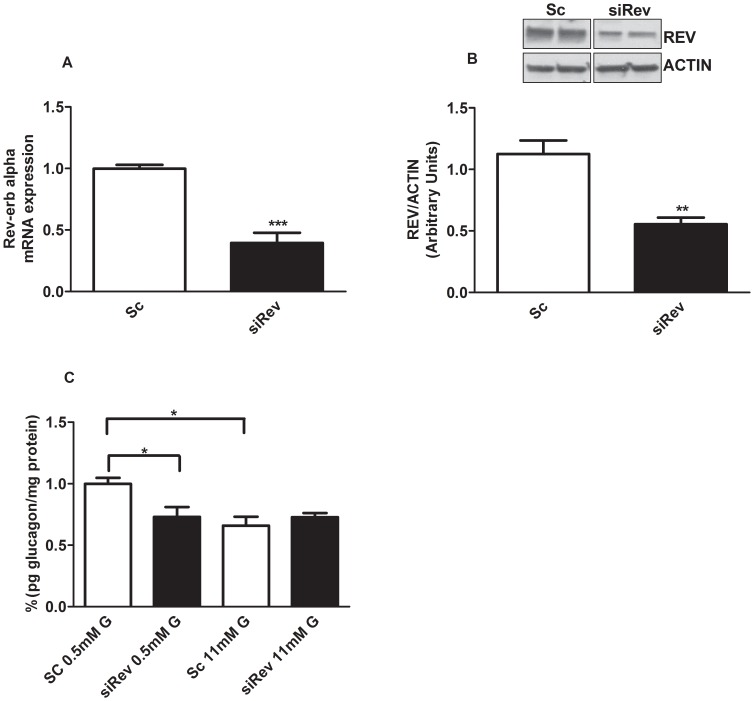
Rev-erb alpha regulates glucagon secretion in alphaTC1-9 cells. (**A**) *Rev-erb alpha* gene expression and (**B**) protein expression from alphaTC1-9 cells treated for 24 hours with 50 nM control scramble siRNA (Sc) and 50 nM Rev-erb alpha siRNA(siRev) (n = 5). (**C**) Glucagon secretion after treatment with Sc and siRev in alphaTC1-9 cells (n = 5–6). *p<0.05, **p<0.01, *** p<0.001 versus Sc. Data are expressed as mean ±S.E.M.

**Figure 3:**
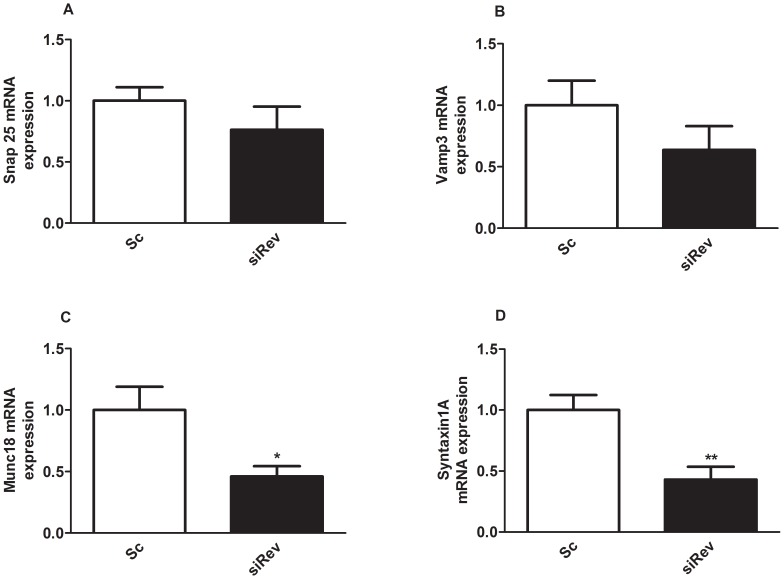
Rev-erb-alpha regulates specific exocytotic genes in alphaTC1-9 cells. (**A**) SNAP 25 gene expression (**B**) Vamp3 gene expression (**C**) Munc18 gene expression (**D**) Syntaxin1a gene expression in alphaTC1-9 cells after treatment with Sc and siRev for 24 hours (n = 6). *p<0.05 **p<0.01, versus control (Sc). Data are expressed as mean ±S.E.M.

To study the functional role of *Rev-erb alpha* in alpha-cells, we used the siRNA technique to down-regulate this gene in mouse alphaTC1-9 cells. Gene silencing efficiency was about 60–70% compared with cells treated with a scramble siRNA (Sc; control siRNA) (Fig. 2A). At these concentrations of siRNA (50 nM) we previously observed no difference in cell viability between scramble and siRNA treated cells 13. The lower *Rev-erb alpha* expression was associated with a decrease in protein level (Fig. 2B). We next investigated whether *Rev-erb alpha* is involved in the regulation of glucagon secretion. Figure 2C shows the glucose effect on glucagon secretion from alphaTC1-9 cells after *Rev-erb alpha* silencing. As previously shown in isolated mouse islets and alphaTC1-9 cells [13], [16], glucagon secretion was stimulated at 0.5 mM glucose whereas secretion was inhibited at 11 mM glucose (Fig. 2C). After silencing the *Rev-erb alpha* gene in alphaTC1-9 cells, glucagon secretion at low glucose concentrations (0.5 mM) was decreased to the same extent as high glucose concentrations (11 mM) (Fig. 2C). No significant differences were observed either on glucagon content or glucagon gene expression after *Rev-erb alpha* silencing (data not shown). Interestingly, when we checked the mRNA expression of exocytotic genes such as Vamp3, Munch18, SNAP25 and Syntaxin1a, there was a down regulation of Munch18 and Syntaxin1a genes (and a tendency to decrease in VAMP3) in alphaTC1-9 cells treated with siRev compared with controls (Fig. 3A–D, respectively). Thus, the decreased glucagon release in cells with down-regulated *Rev-erb alpha* seems more related with changes in the mechanisms that allow the release of glucagon rather than a consequence of an altered glucagon synthesis.

**Figure 4:**
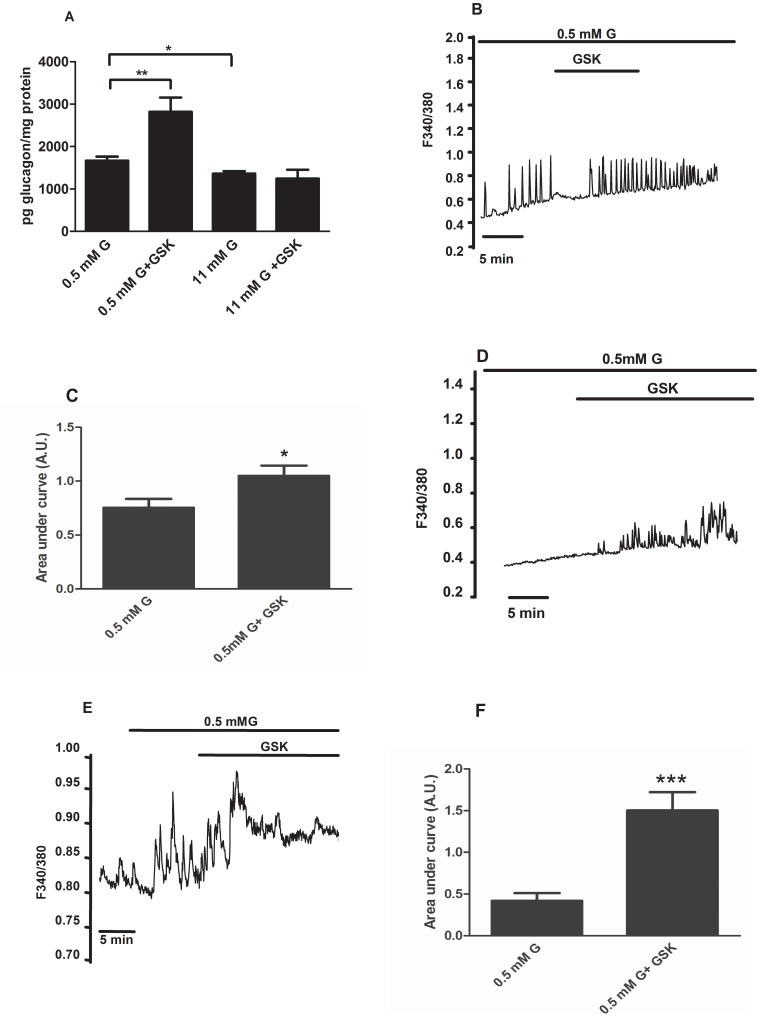
Activation of Rev-erb alpha stimulates glucagon secretion and calcium signals in alphaTC1-9 cells and pancreatic alpha-cells. (**A**) Glucagon secretion in alphaTC1-9 cells in the presence of 0.5 mM, 11 mM glucose and 10 µM of the Rev-erb alpha agonist GSK4112 (n = 7–8). (**B**) Intracellular calcium signals in alphaTC1-9 cells in the presence of 0.5 mM glucose and 10 µM of the Rev-erb alpha agonist GSK4112 (n = 42 cells). (**C**) Area under the curve calculated from experiments illustrated in 4B with alphaTC1-9 cells. (**D**) Intracellular calcium signals in alphaTC1-9 cells in the presence of 0.5 mM glucose and 10 µM of the Rev-erb alpha agonist GSK4112 (n = 24 cells). (**E**) Intracellular calcium measurements in primary mouse alpha cells in the presence of 0.5 mM glucose and 10 µM of Rev-erb alpha agonist GSK4112 (n = 14 cells). (**F**) Area under the curve calculated from experiments illustrated in 4E with primary alpha-cells. *p<0.05, **p<0.01, *** p<0.001. Data are expressed as mean ±S.E.M.

The activity of Rev-erb alpha proteins can be modulated by its natural ligand heme [12], [29] or by the new synthetic Rev-erb alpha agonist GSK4112 and the antagonist SR8278 [30], [31]. To further evaluate the regulatory role of *Rev-erb alpha* in alpha-cell function, we checked the effect of GSK4112 and hemin on glucagon secretion in alphaTC1-9 cells. At 0.5 mM glucose the agonist GSK4112 had a stimulatory effect on glucagon secretion (Fig. 4A) but GSK4112 was not able to revert the inhibitory effects of 11 mM glucose. (Fig. 4A). On the other hand, the natural Rev-erbalpha agonist hemin stimulated glucagon secretion at both 0.5 mM and 11 mM glucose in isolated islets (S 2A). To test the biological effect of hemin, we measured Alas-1 (δ-aminolevulinate synthase 1) a rate limiting enzyme in the heme biosynthetic pathway. As expected the mRNA levels of Alas-1 was decreased in presence of hemin in alphaTC1-9 cells (S 2B).

**Figure 5:**
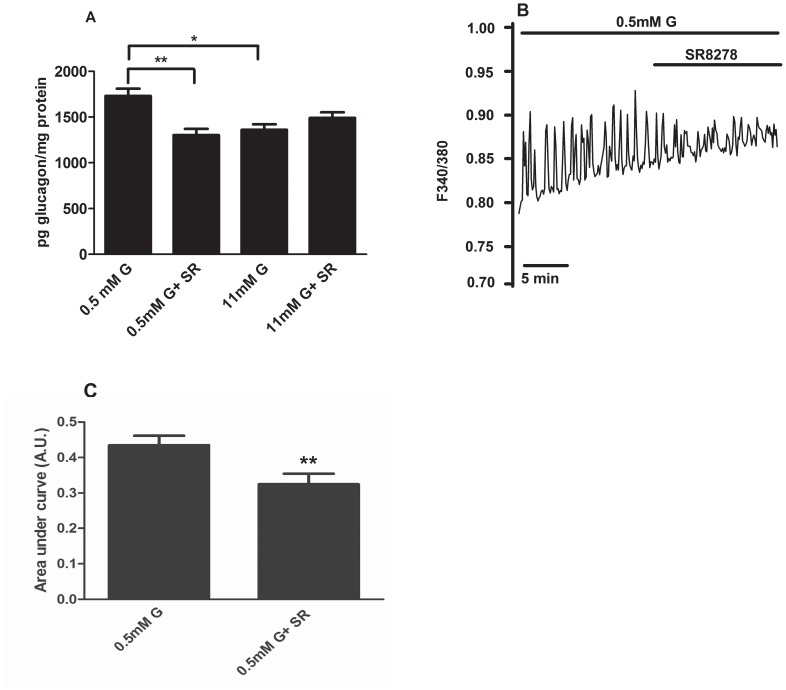
Inhibition of Rev-erb alpha inhibits glucagon secretion and calcium signals in alphaTC1-9 cells and pancreatic alpha-cells. (**A**) Glucagon secretion from alphaTC1-9 cells in the presence with 0.5 mM, 11 mM glucose and 10 µM of the Rev-erb alpha antagonist SR8278 (n = 8). (**B**) Intracellular calcium signals in alphaTC1-9 cells in the presence of 0.5 mM glucose and 10 µM of the Rev-erb alpha antagonist SR8278 (n = 32 cells). (**C**) Area under the curve calculated from experiment 5B. *p<0.05, **p<0.01. Data are expressed as mean ±S.E.M.

The stimulus-secretion coupling of pancreatic alpha-cells is still controversial [20], [23], [32], [33], [34], [35], [36]. However, it is well accepted that glucagon secretion is a calcium-dependent mechanism and changes in calcium signaling regulate the exocytotic process in alpha-cells [34], [35], [37], [38]. Therefore, we checked whether the stimulatory effect of the *Rev-erb alpha* agonist GSK4112 on glucagon secretion could be via modulation of intracellular calcium levels. At low glucose levels (0.5 mM) alphaTC1-9 cells exhibited intracellular calcium oscillations (Fig. 4B). Addition of GSK4112 increased these calcium signals (Fig. 4B) that were statistically significant increased in the area under the curve (Fig. 4C). In some alphaTC1-9 cells that were silent at low glucose concentrations the addition of GSK4112 was able to trigger intracellular calcium oscillations (Fig. 4D). Similar results were achieved in mouse primary alpha-cells. Primary mouse alpha-cells exhibited the characteristic oscillatory calcium pattern at 0.5 mM glucose and addition of GSK4112 increased the signal frequency or transformed calcium oscillations into a sustained pattern (Fig. 4E–F). The synthetic *Rev-erb alpha* antagonist SR8278 had the opposite effect on glucagon secretion and intracellular calcium levels in alphaTC1-9 cells. At 0.5 mM glucose, SR8278 inhibited glucagon secretion and had no additional effect at 11 mM glucose (Fig. 5A). The effect of SR8278 on intracellular calcium was evident by its inhibitory action at 0.5 mM glucose (Fig. 5B and 5C). These results demonstrated that modulation or inhibition of *Rev-erb alpha* regulates glucagon secretion and alpha-cell calcium signalling.

### Glucose Inhibits Rev-erb Alpha Gene Expression via AMPK-Nampt-Sirt1 Pathway

**Figure 6:**
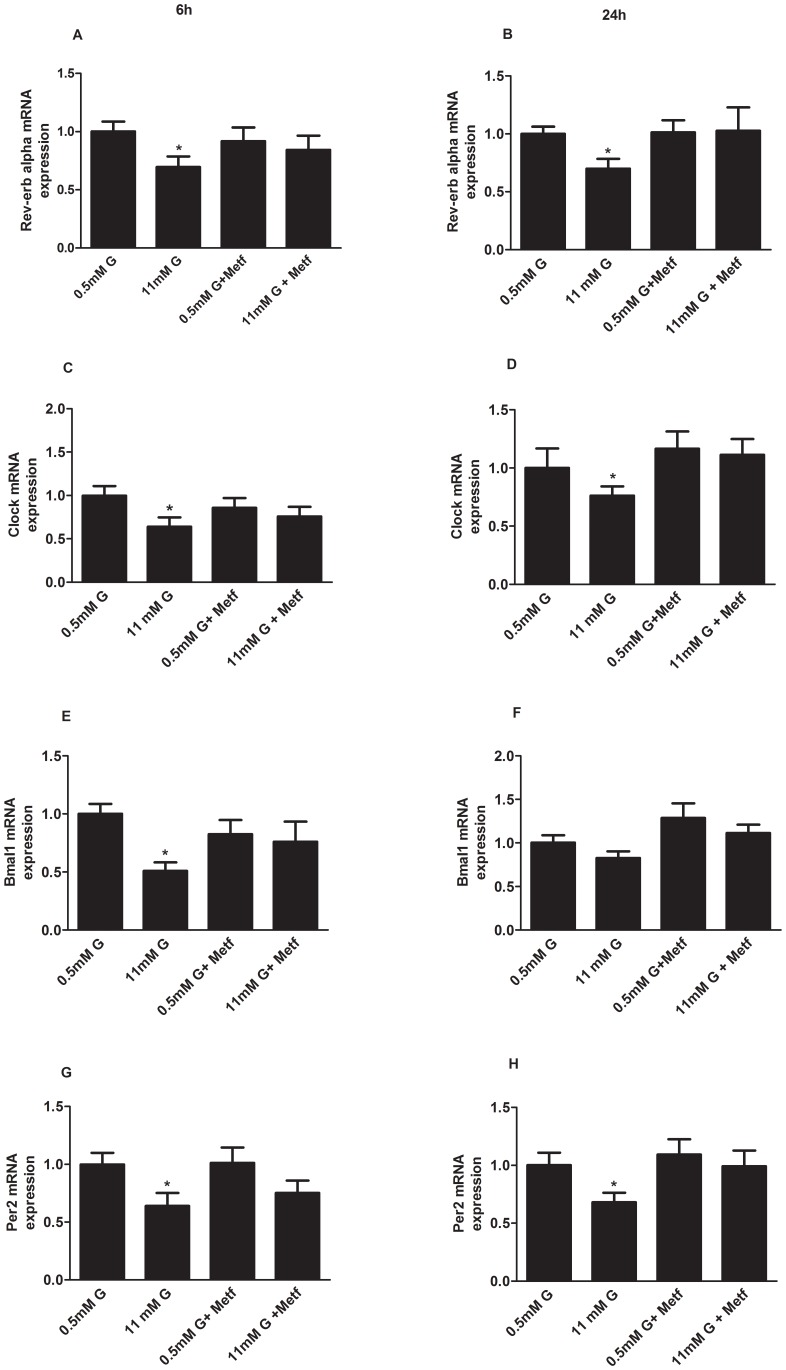
Activation of AMPK prevents glucose inhibition of clock genes in alphaTC1-9 cells. (**A**) *Rev-erb alpha* gene expression in alphaTC1-9 cells treated with 0.5 mM, 11 mM glucose or 500 µM Metformin for 6 hours (n = 5) and (**B**) 24 hours (n = 5). (**C**) *Clock* gene expression in alphaTC1-9 cells treated with 0.5 mM, 11 mM glucose or 500 µM Metformin for 6 hours (n = 5–6) and (**D**) 24 hours (n = 5–6). (**E**) *Bmal1* gene expression in alphaTC1-9 cells treated with 0.5 mM, 11 mM glucose or 500 µM Metformin for 6 hours (n = 5–6) and (**F**) 24 hours (n = 5–6). (**G**) *Per2* gene expression in alphaTC1-9 cells treated with 0.5 mM, 11 mM glucose or 500 µM Metformin for 6 hours (n = 5–6) and (**H**) 24 hours (n = 5–6). *p<0.05; **p<0.01. Data are expressed as mean ±S.E.M.

Since AMPK has been shown to be strongly regulated by glucose in pancreatic alpha-cells, participates in glucagon release [16], and modulates the expression of different clock genes in skeletal muscle, we hypothesized that AMPK might be involved in the mechanism by which glucose regulates *Rev-erb alpha* gene expression. We treated alphaTC1-9 cells for 6 and 24 hours with 500 µM of the AMPK activator Metformin since the AMPK activator AICAR had no effect on alphaTC1-9 cells, as previously shown [16]. As expected, glucose down-regulated *Rev-erb alpha* gene expression at both 6 and 24 hours of treatment (Fig. 6A and B). Remarkably, while AMPK activation by metformin had no effect at 0.5 mM glucose, it prevented the inhibitory effect of 11 mM glucose on *Rev-erb alpha* gene expression (Fig. 6A and B) indicating that AMPK is involved in this process. Similar results were obtained with others clock genes such as Clock (Fig. 6C and D), Bmal1 (Fig. 6E and F) and Per2 (Fig. 6G and H).

**Figure 7:**
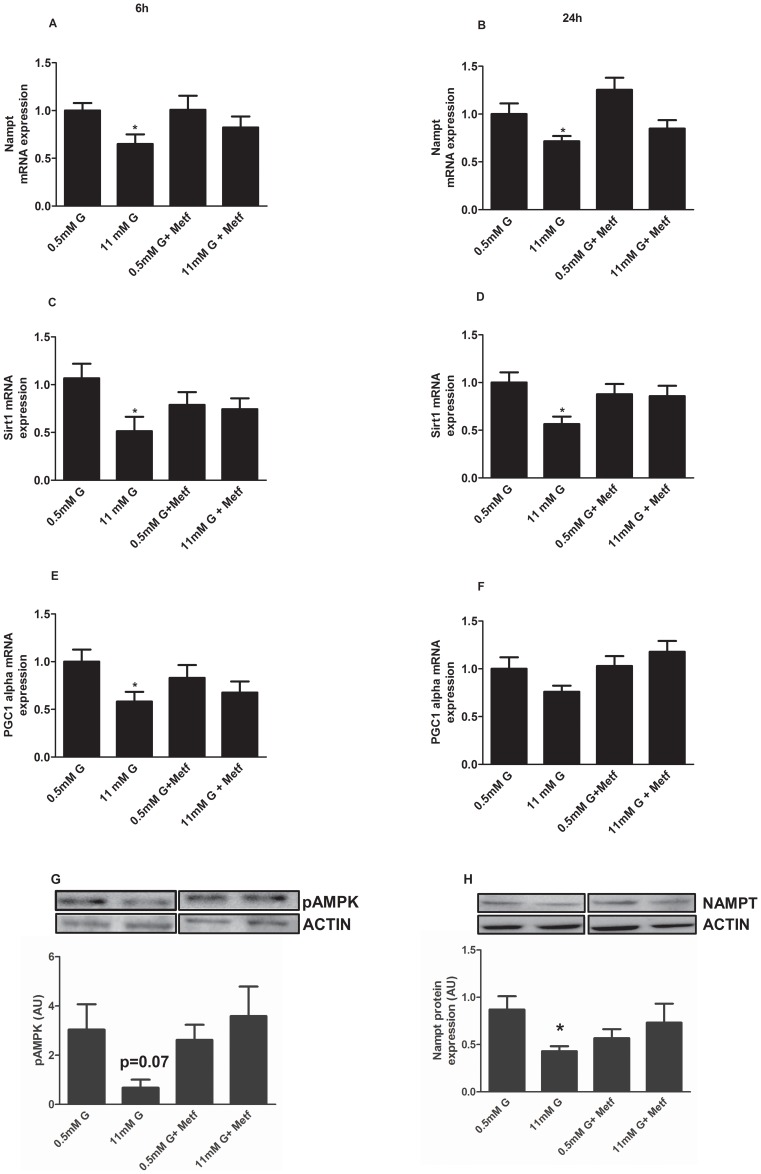
Activation of AMPK prevents glucose inhibition of Rev-erb alpha expression via Nampt-Sirt1 pathway in alphaTC1-9 cells. (**A**) *Nampt* gene expression in alphaTC1-9 cells treated with 0.5 mM, 11 mM glucose or 500 µM Metformin for 6 hours (n = 5–6) and (**B**) 24 hours (n = 5–6). (**C**) *Sirt-1* gene expression in alphaTC1-9 cells treated with 0.5 mM, 11 mM glucose or 500 µM Metformin for 6 hours (n = 5–6) and (**D**) 24 hours (n = 5–6). (**E**) *PGC-1 alpha* gene expression in alphaTC1-9 cells treated with 0.5 mM, 11 mM glucose or 500 µM Metformin for 6 hours (n = 5) and (**F**) 24 hours (n = 5–6). (**G**) AMPK phosphorylation (Thr^172^) in alphaTC1-9 cells treated with 0.5 mM, 11 mM glucose or 500 µM Metformin for 6 hours. (**H**) NAMPT protein expression in alphaTC1-9 cells treated with 0.5 mM, 11 mM glucose or 500 µM Metformin for 6 hours. *p<0.05. Data are expressed as mean ±S.E.M.

It has been demonstrated in white adipose tissue that metformin can control clock genes expression through AMPK-Nampt-Sirt1 pathway [17]. To investigate whether a similar mechanism takes place in pancreatic alphaTC1-9 cells, we next studied this pathway by treating alphaTC1-9 cells for 6 and 24 hours with 500 µM Metformin. Figure 6 shows that 11 mM glucose inhibited Nampt and Sirt1 mRNA levels at 6 hours (Fig. 7A and C) and at 24 hours (Fig. 7B and D). Strikingly, metformin treatment reversed the inhibitory effect of glucose on Nampt (Fig. 6A and B) and Sirt1 (Fig. 7C and D). Consistent with the idea of a glucose-activated AMPK-Nampt-Sirt1 pathway, PGC-1 alpha (peroxisome proliferator-activated receptor gamma coactivator 1-alpha) gene expression (a target of AMPK and Sirt1) was also decreased at high glucose concentrations (Fig. 7E) and AMPK activation by metformin partially prevented this effect (Fig. 7E). The same effect on pAMPK in the Thr^172^ and Nampt protein levels were achieved in alpha alphaTC1-9 cells treated for 6 h (Fig. 7G and 7H) respectively.

### Nampt Inhibition Modulates Sirt1-Rev-erb Alpha Gene Expression and Glucagon Secretion

**Figure 8:**
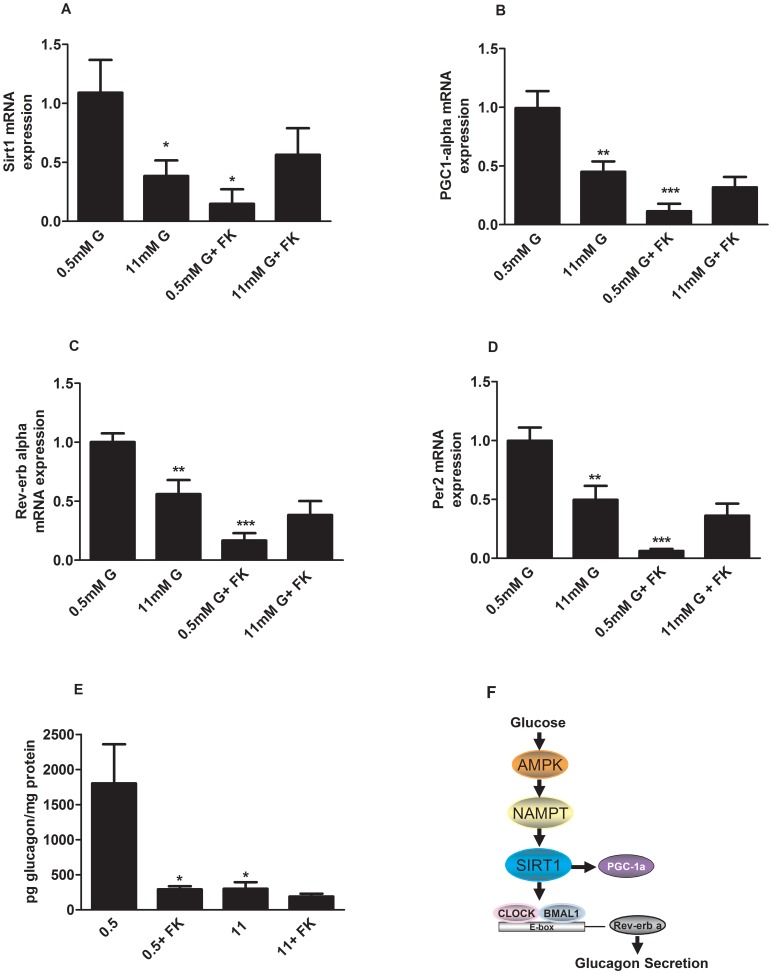
Nampt inhibition leads to decrease in Rev-erb alpha expression and glucagon secretion in alphaTC1-9 cells and pancreatic alpha cells. (**A**) *Sirt1* gene expression in alphaTC1-9 cells treated with 0.5 mM, 11 mM glucose or 500 nM FK 866 for 6 hours (n = 7). (**B**) *PGC-1 alpha* gene expression in alphaTC1-9 cells treated with 0.5 mM, 11 mM glucose or 500 nM FK 866 for 6 hours (n = 6–7). (**C**) *Rev-erb alpha* gene expression in alphaTC1-9 cells treated with 0.5 mM, 11 mM glucose or 500 nM FK 866 for 6 hours (n = 7). (**D**) *Per2* gene expression in alphaTC1-9 cells treated with 0.5 mM, 11 mM glucose or 500 nM FK 866 for 6 hours (n = 7–8). (**E**) Glucagon secretion from mouse pancreatic islets stimulated for 1.5 hour with 0.5 mM, 11 mM glucose or 500 nM FK 866 (n = 6). *p<0.05, **p<0.01, *** p<0.001. Data are expressed as mean ±S.E.M. (**F**) Proposed model for regulation of glucagon secretion via an AMPK-Nampt-Sirt1-Rev-erb alpha mechanism. At low glucose concentrations AMPK is activated which will trigger the Nampt-Sirt pathway increasing Rev-erb alpha expression levels and glucagon secretion. Conversely, high glucose will inhibit AMPK-Nampt-Sirt pathway and consequently Rev-erb alpha expression levels leading to a decrease in glucagon release.

To further examine the role of Nampt-Sirt1 signalling on *Rev-erb alpha* gene expression and glucagon secretion, we used FK866, a highly specific Nampt inhibitor. We treated alphaTC1-9 cells with 500 nM FK866 for 6 hours at low and high glucose concentrations and checked the mRNA expression of Sirt1 and PGC-1 alpha as downstream targets of Nampt. Figure 8A shows that Nampt inhibition by FK866 decreases Sirt1 expression at 0.5 mM glucose while no additional effect was seen at 11 mM glucose. PGC-1 alpha, the downstream target of Sirt1 was also decreased in the presence of FK866 at low glucose concentrations (Fig. 8B). Importantly, Nampt inhibition decreased *Rev-erb alpha* mRNA levels at 0.5 mM glucose whereas at 11 mM glucose when Nampt is already inhibited by glucose, (Fig. 7A and B) this effect was the same as glucose alone (Fig. 8C). Similar results were seen in Per2 mRNA levels in the presence of FK866 (Fig. 8D). Finally, we checked whether inhibition of Nampt affects glucagon secretion in isolated mouse pancreatic islets. As expected, glucose inhibited glucagon secretion at 11 mM glucose (Fig. 8E). Similarly, FK866 inhibitied glucagon secretion at 0.5 mM glucose with no additional effect at 11 mM glucose. These results demonstrate that glucose via AMPK-Nampt-Sirt1 pathway can control *Rev-erb alpha* gene expression and glucagon secretion in pancreatic alpha-cells. We therefore propose that at low glucose concentrations AMPK is activated which will trigger the Nampt-Sirt pathway increasing *Rev-erb alpha* expression levels and glucagon secretion. Conversely, high glucose will inhibit AMPK-Nampt-Sirt pathway and consequently *Rev-erb alpha* expression levels leading to a decrease in glucagon release (Fig. 8F).

## Discussion

The nuclear receptor *Rev-erb alpha* is considered a good candidate to integrate circadian rhythms and metabolism [39], [40]. The regulation of this nuclear receptor was not known until recently and, originally, it was identified as an orphan nuclear receptor due to its canonical domain structure and sequence conservation [41], [42]. However, recent studies have identified the porphyrin heme as a natural ligand for *Rev-erb*s [12], [29]. The present study shows that the clock gene *Rev-erb alpha* is a new intracellular regulator of glucagon secretion, a key process in the control of glucose homeostasis. AlphaTC1-9 cells expressed different clock genes indicating their potential role in this endocrine cell type. Particularly, the *Rev-erb alpha* mRNA levels were found to have intrinsic 24 hours oscillations at low glucose concentrations. The expression levels peaked at ZT6 and the circadian pattern was completely inhibited by high glucose concentrations. These results were confirmed at both the protein level at ZT6 in alphaTC1-9 cells as well as at the mRNA level in sorted mouse primary alpha-cells. In agreement with the present findings, the inhibitory effect of glucose on the expression of Per1 and Per2 mRNA has been shown in rat1-fibroblasts [43]. On the other hand, glucose has no effect on clock gene expression in rat cardiomiocytes [44]. Taken together, our results demonstrate that pancreatic alpha-cells present an intrinsic clock and that the clock gene *Rev-erb alpha* can be induced or repressed according to the glucose levels.

The involvement of *Rev-erb alpha* on glucagon secretion was evident when this gene was down-regulated by siRNA. Reduction of ∼50% in protein and ∼60% in mRNA levels were sufficient to decrease glucagon release at low glucose concentrations. High glucose levels were still capable of reducing glucagon secretion after *Rev-erb alpha* silencing, probably because *Rev-erb alpha* levels were already low at these glucose concentrations or due to other signaling pathways different from *Rev-erb alpha*. Actually, several mechanisms are involved in glucose modulation of glucagon secretion as reported by us and others. [20], [23], [33], [34], [35], [37], [38]. In any case, we identified *Rev-erb alpha* as a new intracellular mediator of glucagon release, whose effects depend on extracellular glucose levels, further supporting the hypothesis that glucose can directly regulate alpha-cell secretion by several pathways [15]. The involvement of *Rev-erb alpha* on glucagon secretion was further confirmed by the stimulatory effect of the *Rev-erb alpha* agonist GSK4112 and its natural ligant hemin and the opposite effect with an antagonist. We have recently demonstrated that *Rev-erb alpha* can regulate insulin secretion probably by downregulation of exocytotic genes in the beta-cell [13], and similar findings have been obtained with other clock genes [14]. In the case of pancreatic alpha-cells, silencing of *Rev-erb alpha* led to a decrease of specific exocytotic genes such as Munch18 and Syntaxin1a, genes that are shown to be involved in the regulation of glucagon exocytosis [45]. Additionally, our results also showed that activation of *Rev-erb alpha* in alpha-cells led to an increase of intracellular calcium concentrations whereas its inactivation decreased calcium signaling. Given the importance of intracellular calcium signals in the regulation of glucagon secretion [20], [24], [33], [35], [36], [38], our results indicate that *Rev-erb alpha* may also regulate glucagon secretion by a calcium-dependent mechanism. Thus, our findings give new insights regarding the regulation of glucagon secretion showing that the clock gene *Rev-erb alpha* modulates glucagon release in a glucose-modulated manner.

The stimulus secretion coupling of alpha-cells and the mechanisms involved in glucagon release are still largely unknown. Among the nutrients that regulate glucagon secretion, glucose is considered one of the most important modulators [20], [38]. However, the intracellular pathway by which glucose inhibits glucagon release is still controversial. It has been proposed that glucose can directly inhibit glucagon secretion independently of paracrine signals from other cell types within the islet of Langerhans [15], [20], [23], [34], [38]. On the other hand, several paracrine mechanisms activated at high-glucose concentrations as a result of beta and delta-cell stimulation have been shown to inhibit glucagon release [15], [33], [35], [36], [46]. Here we show that modulation of *Rev-erb alpha* both acutely (pharmacological modulation of *Rev-erb alpha*) and chronically (inhibition of *Rev-erb alpha* by siRNA) regulates glucagon secretion. The acute effects may involve activation/inhibition of calcium channels by a still unknown mechanism whereas the chronic effect may be due to the effects of glucose on *Rev-erb alpha* gene expression through the AMPK-Nampt-Sirt1 mechanism.

Another important signal that regulates glucagon secretion is AMP-activated protein kinase [16]. It has been reported that glucose inhibits AMPK activity by increasing the ATP/ADP ratio in alphaTC1-9 cells. In addition, pharmacological activation of AMPK with metformin stimulates glucagon secretion whereas inhibition of the kinase leads to decreased glucagon release [16]. In agreement with these previous reports, the use of metformin to activate AMPK in alphaTC1-9 cells led to a decrease in several metabolism and signalling genes. Recently, AMPK has been implicated in the regulation of clock genes in other cell types [5], [17], [47], [48], [49]. Consistent with these reports, we found that AMPK activation by metformin in pancreatic alpha-cells could prevent the inhibitory glucose effect on Rev-erb alpha gene expression and other clock genes such as Clock, Bmal1 and Per2. In white adipose tissue from obese mice, AMPK activation by metformin rescued the defects on clock gene expression via an AMPK-Nampt-Sirt1 mechanism [17]. Our results further support this pathway in pancreatic alpha-cells. The mRNA levels of Nampt, Sirt and PGC-1 alpha were all downregulated by high glucose, a condition where the AMPK is inactivated in pancreatic alpha-cells [16]. AMPK activation by metformin at low glucose had no effect on Nampt, Sirt and PGC-1 alpha mRNA levels because at these glucose levels the AMPK activity is already high in these cells. However, when AMPK was activated by metformin at high glucose, the mRNA levels of Nampt, Sirt and PGC-1 alpha were increased indicating that the inhibitory effect of glucose on these genes was very likely depending on AMPK. Recently, it was demonstrated that AMPK activation increases Sirt1 activity by increasing Nampt expression and NAD^+^ levels [17], [50], [51]. In addition, the AMPK alpha1 and alpha 2 KO mice lack the cyclic expression of Nampt and PGC-1 alpha [52]. Moreover, the NAD^+^ dependent deacetylase Sirt1 binds to the Clock/Bmal1 complex to regulate the expression of clock genes [53], [54]. Consistent with this role of Nampt in the regulation of Sirt1 and clock genes, the highly specific Nampt inhibitor FK866 inhibited Sirt1, PGC-1 alpha, Per2 and Rev-erb alpha mRNA expression in alphaTC1-9 cells and glucagon secretion in mouse pancreatic islets.

In conclusion, we demonstrated here that the clock gene Rev-erb alpha is an intracellular regulator of glucagon secretion in pancreatic mouse alpha-cells. Glucose, by regulating AMPK, may modulate Nampt-Sirt1 signalling leading to the control of Rev-erb alpha gene and glucagon secretion. Strategies to target the Nampt-Sirt1-Rev-erb alpha in pancreatic alpha-cells can be useful for the treatment of hyperglucagonemia present in diabetes.

## Supporting Information

Figure S1 **Clock genes are expressed in AlphaTC1-9 cells.** Expression of (A) Clock (B) Bmal1 (C) Per1 (D) Per2 (E) Cry1 mRNA in alphaTC1-9, hypothalamus and liver. **p<0.01, *** p<0.001 compared to expression levels in alpha TC1-9. Data are expressed as mean ±S.E.M.
(TIF)

Figure S2 **The Rev-erbalpha agonist hemin stimulates glucagon secretion. (A)** Glucagon secretion from mouse pancreatic islets stimulated for 1.5 hour with 0.5 mM, 11 mM glucose or 30 µM Hemin (n = 6). **(B)** Alas-1 gene expression in alphaTC1-9 cells treated with 0.5 mM glucose and 30 µM Hemin for 6 hours (n = 4). *p<0.05, **p<0.01, *** p<0.001. Data are expressed as mean ±S.E.M.
(TIF)
